# *Brevibacterium casei*: A Rare Cause of Peritoneal Dialysis-Associated Peritonitis

**DOI:** 10.1155/crin/3942963

**Published:** 2025-10-15

**Authors:** Veerle Wijtvliet, Katrien Leyssens, Marleen Vanden Driessche, Veerle Matheeussen, Andrea Bertels

**Affiliations:** ^1^Department of Nephrology and Hypertension, University Hospital Antwerp, Antwerp, Belgium; ^2^Laboratory of Experimental Medicine and Pediatrics and Member of the Infla-Med Centre of Excellence, University of Antwerp, Antwerp, Belgium; ^3^Department of Microbiology, University Hospital Antwerp, Antwerp, Belgium; ^4^Laboratory of Medical Microbiology, Vaccine & Infectious Disease Institute (VAXINFECTIO), University of Antwerp, Antwerp, Belgium

**Keywords:** 16S rRNA, *Brevibacterium casei*, MALDI TOF, peritoneal dialysis, peritonitis

## Abstract

Peritonitis is an important complication of peritoneal dialysis (PD), affecting up to 40% of patients at some point of their PD treatment. Here, we describe a case of PD-associated peritonitis due to an unusual pathogen, *Brevibacterium casei*. The patient was treated with intraperitoneal antibiotics, successfully preserving the PD catheter. To the best of our knowledge, only four cases of peritonitis due to *B. casei* have been previously documented worldwide, with catheter preservation achieved in just one other case.

## 1. Introduction

Peritonitis remains an important complication of peritoneal dialysis (PD), frequently leading to a transfer to haemodialysis. It is most often caused by Gram-positive bacteria, accounting for 45%–65% of the cases [1]. Here, we describe a case of PD-associated peritonitis due to an unusual pathogen, *Brevibacterium casei*, a Gram-positive coryneform bacterium identified through matrix-assisted laser desorption/ionisation time-of-flight (MALDI TOF) and confirmed by 16S ribosomal RNA (16S rRNA) gene sequencing. To the best of our knowledge, only four cases of peritonitis due to *B. casei* have been previously documented worldwide [2–5].

## 2. Case Report

A 62-year old Caucasian male on PD was admitted to the emergency department because of increasing abdominal pain since five days. His medical history included Type 2 diabetes, without need for therapy, and epilepsy, for which he was taking levetiracetam as maintenance treatment. The patient had initiated automated peritoneal dialysis (APD) 9 months prior, following nephroureterectomy of his solitary kidney due to a pyelum carcinoma. He had experienced no previous episodes of peritonitis. At the time of hospital admission, the PD prescription comprised 8.5 h of overnight continuous peritoneal dialysis (OCPD) with four cycles, each with a 3-L fill volume of physioneal 1.36/2.27% dextrose bags (tidal at 80%), followed by two cycles of 2.5 L extraneal during the day. Clinical examination revealed a hemodynamically stable patient with a subfebrile body temperature following antipyretic therapy (paracetamol 1 g), along with diffuse abdominal tenderness. There were no signs of exit site or tunnel infection, and the patient denied any manipulation error. Laboratory results were significant for leucocytosis (15.1 × 10^3^/μL) with neutrophilia (13.2 × 10^3^/μL) and C-reactive protein (CRP) of 52 mg/L. The PD effluent was cloudy with a white blood cell count (WBC) of 3085/μL (77.1% polymorphonuclear [PMNs] leukocytes), confirming the diagnosis of peritonitis. After taking two sets of blood culture bottles and inoculating PD fluid in one set as well (BactAlert FA and FN plus, bioMérieux), antibiotic treatment was started with intraperitoneal cefazolin (1.8 g, compatible with 20 mg/kg body weight) and ceftazidime (3 g), along with antifungal prophylaxis (fluconazole 100 mg daily). After one day, the patient's clinical condition further deteriorated with the development of hypotension (96/69 mmHg), increasing abdominal pain, and fever up to 38.6°C. Moreover, after 19.7 h of incubation, the aerobic bottle with PD effluent flagged positive, showing Gram-positive rods with a coryneform shape on Gram stain, and antibiotic therapy was switched to vancomycin intraperitoneally. Subsequent culture showed growth of grey-whitish colonies with a shiny surface, identified as *B. casei* by MALDI-TOF (Bruker), with a maximum log score of 2.4. The identification was later confirmed by Sanger sequencing targeting 500 base pairs of the 16S rRNA gene. Antibiotic susceptibility testing was performed according to EUCAST Breakpoint tables for interpretation of minimum inhibitory concentrations (MICs) and zone diameters, Version 13.0, 2023 guidelines for *Corynebacterium* species [6]. Vancomycin tested sensitive by gradient diffusion test (E-test) with a MIC of 0.25 μg/mL, while penicillin tested resistant (MIC 2 μg/mL). Of note, the anaerobic bottle of PD effluent and the blood cultures remained negative during 5 days of incubation.

**Figure 1:**
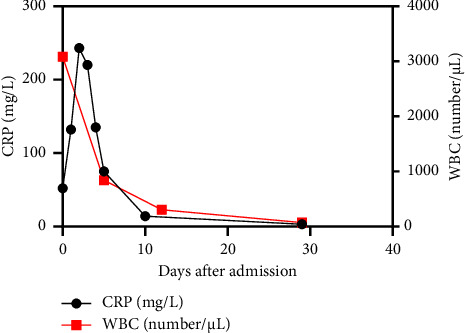
The C-reactive protein (CRP) levels (shown in black) measured in blood, and the white blood cell count (WBC) (shown in red) in peritoneal dialysis effluent, plotted against time from the moment of admission.

According to antibiotic susceptibility testing, intraperitoneal vancomycin was administered continuously for a total 3-week duration, with a target serum trough level of 15 mg/L [4]. After five days of treatment, repeat PD-effluent WBC decreased to 843/μL, and culture remained negative. Subsequent PD-effluent tests at two and four weeks post-treatment showed a further reduction in WBC to 305/μL and 73/μL (Figure 1), respectively, with both cultures remaining negative. Due to the rapid favourable clinical and biochemical improvement, removal of the PD catheter was deemed unnecessary. No relapse of PD-associated peritonitis was observed in the following 7 weeks.

## 3. Discussion

Peritonitis is a common complication of PD, occurring in up to 40% of patients at some point of their treatment. It is associated with considerable morbidity and can lead to membrane failure, catheter loss, transfer to haemodialysis and even death [7]. PD-associated peritonitis is most often caused by Gram-positive bacteria, responsible for 45%–65% of the cases [1]. Here, we report a case of PD-associated peritonitis due to the unusual pathogen *B*. *casei.*

*B. casei* are obligate aerobic, Gram-positive, catalase-producing, immotile, rod-shaped bacteria. They are commonly found in dairy products, such as raw milk or cheese, as well as on human skin. *B. casei* can lead to various infections, including meningitis, salpingitis, cholangitis and peritonitis [8]. Its diagnosis may be challenging, as *Brevibacterium* resembles diphtheroids on Gram stain [4]. While biochemical testing can aid in differentiating *Brevibacterium* from other organisms, 16S rRNA gene sequencing is considered the gold standard for definitive species identification [9]. Despite being sensitive to vancomycin, three cases of recurrent PD peritonitis have already been reported [2, 3, 5]. So far, in addition to our case, the PD catheter could only be salvaged in one other case (Table 1) [4].

Current treatment guidelines recommend a 2-week course of antibiotics for Gram-positive peritonitis, except 3 weeks for *Staphylococcus* and *Enterococcus* infections [7]. Based on our case and the case of Roy et al., an extended treatment duration of 3 weeks may be required for *B. casei* to ensure eradication and prevent relapse [4]. Most *Brevibacterium* isolates are susceptible to vancomycin, which remains the first-choice antibiotic. Antimicrobial resistance of more than 50% has been observed for trimethoprim/sulfamethoxazole, clindamycin and first-line beta-lactams [10].

In conclusion, when managing PD peritonitis, inoculating PD fluid in blood culture bottles is essential for accurately identifying unexpected pathogens, determining their susceptibilities and guiding tailored antibiotic therapy.

## Figures and Tables

**Table 1:** Summary of case reports published on *Brevibacterium casei* peritonitis.

First author	Year	Location	Age/sex	Dialysis modality	Treatment^∗^	Outcome	Modality changed
Poesen et al. [3]	2012	Belgium	37/M	CCPD	IP vancomycin (2 weeks)	Delayed recovery with catheter removal	Switch to HD
Althaf et al. [2]	2014	Saudi Arabia	33/F	CCPD	IP ceftazidime and IP cefazolin (2 weeks)	Delayed recovery with catheter removal	Switch to HD
Roy et al. [4]	2022	USA	63/M	CCPD	IP vancomycin (3 weeks)	Direct recovery with catheter salvage	No
Roger et al. [5]	2024	France	75/M	CAPD	IP vancomycin (3 weeks)	Delayed recovery with catheter removal	Timely switch to HD, followed by resumption of PD

*Note:* F, female; HD, haemodialysis; IP, intraperitoneal; M, male.

Abbreviations: CAPD, continuous ambulatory peritoneal dialysis; CCPD, continuous cycler peritoneal dialysis; PD, peritoneal dialysis; USA, United States of America.

^∗^After initial identification of *B. casei*.
